# Analysis of Multiple Health Risky Behaviours and Associated Disease Outcomes Using Scottish Linked Hospitalisation Data

**DOI:** 10.3389/fpubh.2022.847938

**Published:** 2022-07-11

**Authors:** Damilola Olajide, Barbara Eberth, Anne Ludbrook

**Affiliations:** ^1^Research Design Services East Midlands, School of Medicine, Queens Medical Centre, University of Nottingham, Nottingham, United Kingdom; ^2^Newcastle University Business School, Newcastle upon Tyne, United Kingdom; ^3^Health Economics Research Unit, University of Aberdeen, Aberdeen, United Kingdom

**Keywords:** multiple health behaviours, Scottish adults, linked Scottish health survey, recursive multivariate probit model, lifestyle specific diseases

## Abstract

**Background:**

Disease incidence and premature deaths tend to be influenced by multiple health risky behaviours, including smoking, excessive alcohol consumption and unhealthy diet. Risky behaviours tend not to be independent and may have a multiplicative effect on disease incidence and healthcare cost. Thus, understanding the interrelationship between health behaviours and their effect on health outcomes is crucial in designing behavioural intervention programmes.

**Objective:**

To examine the interrelationship between health risky behaviours and associated disease outcomes amongst Scottish adults.

**Methods:**

We use hospitalisation episode data from the Scottish Morbidity Records, (SMR), that have been administratively linked to Scottish Health Surveys (SHeS) respondents with target disease defined by relevant ICD9 and 10 codes. We apply a recursive multivariate probit model to jointly estimate the health risky behaviours and disease incidence to adequately control for unobserved heterogeneity. The model is estimated separately by gender.

**Results:**

Modelling health risk behaviours and disease incidence equations independently rather than jointly may be misleading. We find a clear socioeconomic gradient predicting health risky behaviours and the results differ by gender. Specifically, smoking appears to be a key driver of other health risky behaviours. Current smokers are more likely to be drinking above the recommended limit, physically inactive, and eating inadequate diet.

**Conclusions:**

Interventions targeting current smokers to quit could spillover to other behaviours by reducing excessive drinking, improve physical activity and adequate diet. Thus, improvements in one behaviour may increase the likelihood of adopting other healthier lifestyle behaviours.

## Introduction

Health risky behaviours, including smoking, excessive alcohol consumption, physical inactivity and inadequate diet have a direct impact on hospital care demand. These risky behaviours are major factors associated with chronic disease incidence, including heart diseases, stroke, type 2 diabetes, obesity, and certain types of cancer; and a major cause of death. For example, 1.7 million or 2.8% of total number of deaths worldwide are attributable to low fruit and vegetable consumption, and considered amongst the top 10 risk factors for global mortality. Low fruit and vegetable intake was associated with around 14% of cancer and gastrointestinal related deaths and about 9% of stroke related deaths (1). In 2009, smoking attributable admissions in Scotland amounted to 56,153 and 13,044 deaths (2), whilst the prevalence rate was 24% on average between 2009/2010 amongst 16–64 year olds based on self-reported smoking status (3).

Chronic disease incidence resulting from risky behaviours presents a significant economic burden to health care systems and greater costs to the society. Scarborough et al. (4) estimated the economic burden of ill health due to diet, physical inactivity, smoking, alcohol and obesity individually in the UK. The study found that in 2006/07, the cost of ill health associated with poor diet amounted to £5.8 billion, physical inactivity £0.9b, smoking £3.3b and alcohol-related £3.3b, whilst overweight and obesity was £5.1b.

Previous studies examining links between these behavioural risk factors and subsequent hospital admissions and death tend to assume an independence relationship between the health risky behaviours. Using a unique Scottish data resource (linked Scottish health survey), Hanlon et al. (5) used the 1998 version of the linked data to analyse the independent association between each behavioural factor and subsequent hospital admissions. In Lawder et al. (6), the same authors retrospectively extended their analysis by including the 1995 linked data to also predict deaths. Both studies found that each health behaviour was independently associated (positively or negatively) with the probability of subsequent hospital admissions and death. For example, not meeting the daily fruit and vegetable five-a-day recommendation was associated with increased risk of admissions and death. Smoking was found to have the highest hazard ratio of all the behavioural factors. In their study limitations however, the authors suggested that future research should investigate associations between risk factors and hospital admissions that are disease-specific diagnoses.

Multiple health risky behaviours tend to occur concurrently or complement each other [e.g., (7, 8)], implying a multiplication of associated disease burdens on health and productivity, pressure on health care systems both in terms of disease incidence and associated costs of hospitalisation (9–11). Moreover, cardiovascular diseases tend to be associated with more than a single health behaviour, suggesting potential intercorrelation between these risky behaviours. However, few studies have been undertaken to understand the patterns that such interactions between health risky behaviours and associated health outcomes might take.

This study examined the interrelationships between risky health behaviours and associated disease outcomes, using large linked health care data on Scottish adults (the linked Scottish health survey). The linked Scottish health survey (linked SheS) links the baseline information on risky behaviours and other characteristics in three waves of the Scottish Health Survey [i.e., (12–14)] to subsequent hospital admissions that were behaviour-specific diagnoses up to 2013. Linking behaviour-specific diagnoses provided a way to approximate causality between health behaviours and disease incidence.

To consistently examine the interrelationships, a recursive multivariate probability (probit) model was adopted. This model jointly estimated a disease incidence equation and a reduced form equation for each of the health risky behaviours; smoking, excess alcohol consumption, physical inactivity, and inadequate diet. The model enabled us to assess the association of demographic, socioeconomic, and other factors with risky lifestyle choices, and investigated their influence on the probability of having a related disease incidence. The modelling approach also allowed us to account for (i) the correlation of unobserved characteristics that may impact both disease risk and risky health behaviours, and (ii) the potential endogeneity of the risky health behaviours to disease incidence. The model was estimated separately for female and male samples.

The model statistic showed that modelling health risk behaviours and disease incidence equations independently rather than jointly may be misleading. The estimation results showed a clear socioeconomic gradient predicting health risky behaviours. Specifically, smoking appeared to be a key driver of other health risky behaviours. Current smokers were more likely to be drinking above the recommended limit, physically inactive, and eating inadequate diet. These results differ by age and gender. Undertaking health risky behaviours decreases with age. Also, males were more likely than females to undertake health risky behaviours: smoking, excessive drinking and inadequate diet, longer in their lives.

The findings suggest that targeting current smokers to quit in interventions to promote multiple healthier lifestyles may spill over to other lifestyles by reducing excessive drinking, improve physical activity and adequate diet amongst individuals with multiple health risky behaviours. This implies that, improvements in one behaviour may increase the likelihood of adopting other healthier lifestyles.

The remainder of the paper is organised as follows. Section on Materials and Methods provides a data description and outlines the estimation model. Results are presented next followed by the Discussion.

## Materials and Methods

### Data

#### The Linked Scottish Health Survey Dataset

The hospitalisation episodes data used in this study were sourced from the Scottish Morbidity Records (SMR) that were administratively linked to respondents to Scottish Health Surveys (SHeS) by the Information Services Division (ISD) of the National Health Services (NHS) Scotland. The SHeS is a national representative survey of individuals living in private households in Scotland. It collects a wide range of information on individual respondents, including demographic, socioeconomic, environmental, self-reported health, and health behaviours.

The SMR system other the other hand, records details of all hospitalisation episodes in Scottish NHS hospitals for each respondent in the SHeS who consented to having their information passed onto the ISD. Each episode record contains primary and secondary diagnoses identified by disease-specific International Classification of Diseases ICD9 and ICD10 codes, date of admission and discharge. The SMR is routinely linked to cancer and death registrations, covering the period 1981 to the present. Permission from the Privacy Advisory Committee of the ISD was granted to access the linked SHeS datasets.

#### Health Risky Behaviours and Associated Disease Outcome Variables

The health risky behaviours considered were smoking, alcohol consumption over the limit, physical inactivity, and inadequate diet. All were measured as binary variables in this study. The surveys collected a comprehensive range of data pertaining to alcohol consumption from respondents aged 16 and older, allowing the derivation of weekly units of alcohol consumption. From this, we derived an indicator variable taking a value of one if the individual reported drinking regularly over the moderate limit, and zero otherwise. The drinking limits at the time of the survey were 1 to 14 units for women and 21 units for men (15)^1^.

For smoking, individual respondents aged 16 and older were asked if they “smoke cigarettes regularly nowadays”. Respondents answered either “Yes” or “No”. Thus, the binary “Smoking” variable took a value of one if respondents answered “Yes”; and zero otherwise. For physical inactivity we generated the binary variable “Inactive” taking a value of one if the individual indicated to have spent no time in sporting activities 28 days prior to interview; and zero otherwise.

We proxy diet by use of the number of portions of fruit and vegetables consumed by each respondent over the 24 h period prior to interview. Information on fruit and vegetables include the following food types: fresh, frozen and canned vegetables; vegetables in composites (vegetable lasagne); pulses; salads; fresh, frozen or canned fruit; dried fruit; and fruit in composites (apple pie). Portion size was defined as 80 g and visualised to respondents in tablespoons, cereal bowls and slices. The number of portions of fruits and vegetables were then summed for each respondent to estimate the number of portions of fruits and vegetables consumed in the day prior to interview. The policy recommendation at the time of the survey was five fruit or vegetable portions for adults per day (16) and this has not changed. We derived a binary indicator variable taking the value of one if the individual did not meet the recommended portion size per day; and zero otherwise.

Diseases associated with these health behaviours include cancers, heart diseases, stroke, hypertension, asthma, obesity as a disease, peripheral vascular diseases (PVC), and others identified by the relevant ICD9 and ICD10 codes defining the obesity-, smoking-, and alcohol-related diseases.

Previous evidence showed obesity-related diseases to be associated with diet and physical inactivity [e.g., (16–18)]. We observed the ICD9 and ICD10 codes identifying important diseases such as cancers, stroke and heart diseases tend to overlap, as the same codes identify different behaviour-related diseases. Therefore, disease incidence was defined as the presence of any disease-specific ICD9 and ICD10 codes in any of the diagnosis codes recorded in the data, either as primary diagnosis or cause of death. We used the survey interview date, hospital admission date for an episode, and discharge date to determine when a behaviour-related disease incidence occurred, either pre-survey, post-survey, or both.

A *pre-survey* incidence occurred if the individual was discharged on or before the interview date, whilst an incidence occurred *post-survey* if the individual was admitted on or after the survey interview date. We focus on the first incidence of a disease post-survey to derive the binary disease outcome variable (SMRpost), taking the value one if the individual had the first incidence of a behaviour-related disease *post-survey*; and zero otherwise. We expected each of the risk behaviours to be positively associated with post survey disease incidence.

#### Other Variables

Other variables included in the model as determinants of risky health behaviour and disease incidence are individual demographic, socioeconomic, environment, and self-reported health-related factors and disease history as measured by the presence of a disease incidence pre-survey (SMRpre). Demographic factors include gender, age and marital status. Age enters the model non-linearly via a squared age term in addition to age. Marital status was categorised into three binary variables: Single; Married or co-habiting; and Divorced, widowed, or separated.

The socio-economic characteristics were educational attainment and occupational status. Educational attainment was categorised into 5 groups; no educational qualifications; O-level or equivalent; A-level or equivalent; Further education or equivalent; and University degree or equivalent professional qualifications. Occupational status was categorised into Unskilled; Partly-skilled manual; Skilled manual; Skilled non-manual; Intermediate/managerial; and professional. We used area deprivation to proxy the individual's living environment, measured by the Scottish Index of Multiple Deprivation (SIMD) which is the Scottish Executive's official measure for identifying small area concentrations of multiple deprivations across Scotland (19). SIMD was categorised into ordered quintiles where SIMD51 = 1 indicated the least deprived and SIMD55 = 1 indicated the most deprived quintile.

Self-reported general health was categorised into three binaries: “Very good” (reference group), “Good”, “Fair”; and “Bad/Very bad” health. The remaining self-reported health related factors were binary variables capturing “precondition” including; presence of a medical pre-condition (e.g., IHD, Type 2 diabetes, treatment for high blood pressure, limiting and non-limiting long-standing illness, hospital attendance for a cardiovascular disease (CVD), etc.,), and the presence of the first incidence of a behaviour-related disease incidence pre-survey. Both variables were intended to capture the effect of medical history including pre-survey hospitalisations.

#### Patterns of Risky Behaviours, Disease Incidence and Individual Characteristics

Restricting attention to adults aged 16 years and older, and excluding respondents whose status within the SMR was unknown at the end of the study period, our final sample consisted of *N* = 20,751 individuals. Descriptive statistics in Table 1 showed health risk behaviours patterns and disease incidence. Figures were presented for the whole sample (ALL) and each risky behaviour separately:

**Table 1:** Descriptive statistics of risky behaviours, disease outcome, and other characteristics (*N* = 20,751).

	**All**		**Smoking*^a^***		**Drinking*^b^***		**Inactivity*^c^***		**Diet*^d^***		**SMRpost*^e^***	
**Variable**	**Mean**	**sd**	**Mean**	**sd**	**Mean**	**Sd**	**Mean**	**sd**	**Mean**	**sd**	**Mean**	**Sd**
**Risky behaviours and SMRpost:**												
Smoking	0.365	0.482			0.433	0.495	0.378	0.485	0.386	0.487	0.418	0.493
Drinking above limit	0.241	0.427	0.285	0.451			0.214	0.410	0.236	0.425	0.205	0.404
Inactive	0.500	0.500	0.513	0.500	0.450	0.498			0.506	0.500	0.649	0.477
Inadequate diet	0.907	0.290	0.958	0.201	0.891	0.311	0.921	0.269			0.930	0.256
SMRpost	0.285	0.451	0.326	0.469	0.242	0.429	0.371	0.483	0.291	0.454		
**Characteristics:**												
Gender (male)	0.455	0.498	0.461	0.499	0.628	0.483	0.429	0.495	0.461	0.499	0.470	0.499
Age	46.15	16.11	43.00	14.76	43.17	15.11	51.61	15.89	45.754	16.079	55.81	14.91
Age squared	2,389.3	1,555.1	2,067.3	1,338.7	2,091.6	1,383.2	2,916.5	1,640.7	2,351.97	1,545.18	3,337.6	1,604.9
**Marital status:**												
Single	0.220	0.414	0.264	0.441	0.273	0.445	0.173	0.378	0.225	0.418	0.152	0.359
Married or cohabiting	0.585	0.493	0.502	0.500	0.570	0.495	0.583	0.493	0.578	0.494	0.541	0.498
Divorced/wid./separated	0.195	0.396	0.234	0.423	0.157	0.364	0.244	0.429	0.197	0.398	0.307	0.461
**Educational qual.:**												
None ***^f^***	0.358	0.479	0.405	0.491	0.264	0.441	0.506	0.500	0.367	0.482	0.542	0.498
education2 ***^g^***	0.215	0.411	0.255	0.436	0.223	0.416	0.175	0.380	0.223	0.416	0.183	0.387
education3 ***^h^***	0.126	0.331	0.108	0.310	0.155	0.362	0.103	0.304	0.126	0.331	0.086	0.281
education4 ***^i^***	0.136	0.342	0.126	0.332	0.155	0.362	0.101	0.301	0.135	0.342	0.081	0.273
Degree /professional	0.150	0.357	0.084	0.277	0.192	0.394	0.106	0.307	0.132	0.339	0.088	0.284
education6 (missing)	0.016	0.125	0.022	0.148	0.012	0.108	0.010	0.100	0.017	0.129	0.019	0.136
**Occ. social status:**												
Unskilled/others	0.063	0.242	0.089	0.284	0.044	0.206	0.068	0.252	0.067	0.250	0.094	0.292
Partly skilled manual	0.141	0.348	0.188	0.391	0.128	0.335	0.155	0.362	0.148	0.355	0.168	0.374
Skilled manual	0.249	0.433	0.282	0.450	0.270	0.444	0.287	0.452	0.256	0.436	0.275	0.446
Skilled non-manual	0.187	0.390	0.172	0.378	0.157	0.364	0.176	0.381	0.188	0.391	0.164	0.370
Intermediate/managerial	0.271	0.445	0.202	0.401	0.305	0.461	0.250	0.433	0.257	0.437	0.235	0.424
Professional	0.056	0.229	0.029	0.167	0.072	0.258	0.038	0.190	0.049	0.217	0.035	0.184
Skill (missing)	0.033	0.179	0.038	0.191	0.024	0.152	0.026	0.160	0.035	0.184	0.028	0.166
**Deprivation:**												
Least deprived	0.172	0.377	0.111	0.315	0.204	0.403	0.131	0.338	0.165	0.371	0.116	0.320
Deprived = 2	0.196	0.397	0.145	0.352	0.197	0.398	0.178	0.383	0.189	0.392	0.162	0.369
Deprived = 3	0.211	0.408	0.200	0.400	0.197	0.398	0.219	0.414	0.209	0.407	0.214	0.410
Deprived = 4	0.211	0.408	0.241	0.428	0.206	0.405	0.227	0.419	0.216	0.412	0.237	0.425
Most deprived	0.210	0.408	0.303	0.460	0.196	0.397	0.245	0.430	0.220	0.414	0.271	0.444
**Self-Assessed Health:**												
Very good	0.338	0.473	0.262	0.440	0.350	0.477	0.266	0.442	0.332	0.471	0.192	0.394
Good	0.393	0.489	0.404	0.491	0.418	0.493	0.367	0.482	0.395	0.489	0.342	0.474
Fair	0.197	0.398	0.237	0.426	0.180	0.384	0.249	0.433	0.202	0.401	0.300	0.458
Bad/very bad	0.071	0.257	0.097	0.296	0.052	0.223	0.118	0.323	0.072	0.259	0.167	0.373
Pre-conditions ***^j^***	0.379	0.485	0.334	0.472	0.336	0.472	0.518	0.500	0.370	0.483	0.599	0.490
Disease pre-survey (SMRpre) ***^k^***	0.072	0.259	0.058	0.233	0.051	0.220	0.111	0.314	0.070	0.256	0.188	0.391

a *Smoking, current smoker*.

b *Drinking, alcohol consumption above the recommended limit (underlimit is 1–14 units for females and 21 units for male=)*.

c *Inactivity, undertaking no physical activity*.

d *Diet, Inadequate diet—not meeting the recommended portions of fruits and vegetable per day*.

e *SMRpost,1 if the individual has a behaviour related diseases incidence post-survey*.

f *No education qualification*.

g *Low level, School leaving, CSE, etc*.

h *Low mid. level, SVQ, “A” levels, OND, etc*.

i *Upmided, C&G, HNC, HND, etc*.

j *Preconditions include IHD, Type 2 diabetes, treatment for high blood pressure, limiting and non-limiting long-standing illness, hospital attendance for a cardiovascular disease, etc*.

k *SMRpre, 1 if the individual has a behaviour related diseases incidence post-survey*.

At the time of interview, 50% of the total sample were physically inactive (*N* = 10,157), 36.5% reported to be current smokers (*N* = 7,581), 90.7% were failing the recommended five a day (inadequate diet) (*N* = 18,829), and 24.1% were drinking above moderate alcohol limits (*N* = 4,991). Cross-tabulations across risky behaviours (not in Table 1 but available from the authors) show that 9.6% of respondents had at least one other risky behaviour (*N* = 1,997), whilst 4.7% had all the four health risky behaviours considered in this study (*N* = 980). Moreover, 28.5% of the sample experienced a behaviour-related disease incidence post-survey (*N* = 5,903). Within this sub-sample, 65% were physically inactive, 42% were smokers, 93% had an inadequate diet, and 21% consumed alcohol regularly above the moderate limit.

The average age in the sample was 46 years. However, current smokers, excessive drinkers, and individuals taking inadequate diet were slightly younger whilst the physically inactive were on average older. Those who experienced a disease incidence were on average 56 years old. Thus, we expected age to impact differentially across risk behaviours and disease incidence. In terms of gender, 46% percent of the whole sample were men. The proportion of males undertaking at least one risky behaviour was generally above 42% in the risk behaviour sub-groups, with the highest being drinking above the recommended limit (63%). Within the groups of individuals with a disease incidence 47% were men.

Regarding marital status, 58.5% of the sample were married or cohabiting. Across the health risk behaviours, married and cohabiting individuals were in the majority, and similarly for those with a disease incidence. Over a third (36%) of the sample had no formal educational qualification. Within the risk behaviour sub-groups, respondents with no educational qualification were more likely to undertake each of the risky behaviours: smoking (40.5%), excessive drinking (26.4%), physically inactive (50.6%), and inadequate diet (36.7%), and to have a disease incidence (54.2%). Thus, we expected the probability of undertaking a risky behaviour to decrease with education level.

With respect to occupational social status, a greater proportion of the total sample were in skilled manual and intermediate/managerial occupations, whilst individuals in the unskilled and professional categories were generally <10%. We observe this pattern across all risk behaviours and disease incidence. Regarding deprivation quintiles, we expected the probability of undertaking each of the risky behaviours to decrease with lower levels of deprivation. This was largely because, individuals in the most deprived group were generally lower than a third of the total sample. In the most deprived group were; almost a third (30.3%) of smokers, almost a quarter (24.5%) of physically inactive, more than one-in-five (22%) of those taking inadequate diet, and over a quarter (27.1%) of those with a disease incidence.

For health-related characteristics, 37.9% reported a health-related pre-condition. Around a third of current smokers (33.4%) and excessive drinkers (34%) reported a pre-condition. respectively Almost two-in-five (37%) as well as over half (52%) of the total sample of those with a pre-condition were undertaking inadequate diet, and physically inactive, respectively. Furthermore, a majority of respondents with a pre-condition (60%) experienced a disease incidence. Therefore, we expected pre-condition to increase the probability of disease incidence but might decrease the probability of undertaking risky behaviours. The majority of respondents in the total sample reported their general health to be “Very good” (33.8%) or “Good” (39.3%), and a similar pattern prevailed across the risky behaviour and disease incidence sub-samples.

Finally, 7.2% of the total sample experienced a disease incidence *pre-survey*; 6% of current smokers; 5% of excessive drinkers; 11% of the inactive; and 7% of those with inadequate diet. Of those with a disease incidence *post-survey*, 19% also reported a pre-survey disease incidence. Thus, we expected the probability of a disease incidence post-survey to increase with health-related respondent characteristics.

### Model

The multivariate probit model with endogenous variables belongs to the general class of simultaneous equations system and a generalisation of the bivariate probit model [e.g. (20, 21)]. Previous studies that applied the recursive multivariate probit model in health economics include Contoyannis and Jones (22), Balia and Jones (23), Di Novi (24) and Schneider and Schneider (25, 26). We also considered this model as appropriate to address our study objective. Firstly, the model explicitly accounts for correlations between unobserved characteristics that may determine both health risky behaviours and disease incidence. That is, the random components in the health risky behaviour equations were allowed to be freely correlated with the random component in the structural disease incidence equation. Secondly, the approach accounted for the potential endogeneity bias between health risk behaviours and disease incidence.

In our application, the recursive structure of the model was built around four reduced-form risk behaviour equations and a disease incidence structural equation with risk behaviour variables as predictors. For each latent dependent variable $y_{}^{*}$ for the *i*th individual and *k*th health risk behaviours [*k* = smoking (*smk*), excessive drinking (*drk*), physical inactivity (*inact*), inadequate diet (*diet*) and disease incidence (*dis*)];


(1) $$\begin{array}{r} y_{ik}^{*}\;=\;\beta_{k}^{{}^{\prime }}X_{ik}\;+\;\varepsilon_{ik} \end{array}$$

Let $y_{ik}=1\;\text{if}\;y_{ik}^{*}>0;\;\text{and}\;\text{0}\;\text{otherwise}$, the corresponding binary observed outcome *y* for each of the *k*th health risk behaviours and disease incidence can be stated as:


(2) $$\begin{array}{l} y_{i,smk}\;=\;{\beta^{\prime }}_{smk}X_{i,smk}\;+\;\varepsilon_{i,smk},\\ y_{i,drk}\;=\;{\beta^{\prime }}_{drk}X_{i,drk}\;+\;\varepsilon_{i,drk},\\ y_{i,inact}\;={\beta^{\prime }}_{inact}X_{i,inact}\;+\;\varepsilon_{i,inact},\\ y_{i,diet}\;={\beta^{\prime }}_{diet}X_{i,diet}\;+\;\varepsilon_{i,diet},\\ \text{ and}\\ y_{i,dis}\;=\;\theta_{smk}y_{i,smk}\;+\;\theta_{drk}y_{i,drk}\;+\;\theta_{inact}y_{i,inact}\;+\;\theta_{diet}y_{diet}\\ \;+{\beta^{\prime }}_{dis}Z_{i,dis}\;+\;\varepsilon_{i,dis} \end{array}$$

where ε_*ik*_were the error terms distributed as multivariate normal, each with a mean of zero and a variance-covariance matrix of the cross-equation error terms, *V*, which has values of 1 on the leading diagonal; and correlations ρ_*jk*_ = ρ_*kj*_ off-diagonal elements for *j, k* = 1, …, *M* and *j* ≠ *k*. The parameter ρ_*jk*_ to be estimated, provides the correlations between the error terms of equations *j* and *k*, measuring the extent to which unobserved characteristics of individuals influence their risky behaviour and associated disease incidence. *X*_*ik*_ and *Z*_*i, dis*_ were vectors of explanatory variables in each of the risky behaviour equations and the disease incidence equation, respectively, whilst β_*k*_ and θ_*k*_ were parameter vectors to be estimated.

In application, the vector of explanatory variables in *X* differed from those in vector *Z* (i.e., *X* ≠ *Z*) due to exclusion restrictions on the structural equation. Equation (2) has the structure of a Zellner (27) Seemingly Unrelated Regression model, except that the dependent variables *y*_*ik*_, are binaries and the explanatory variables need not be the same in all equations. The parameter vectors, θ_*smk*_, θ_*drk*_, θ_*inact*_, θ_*diet*_, β_*dis*_ and ρ were estimated using the MVPROBIT programme developed by Capellari and Jenkins (28) and implemented in STATA.

Finally, in addition to the non-linear functional form of the model, identification of the parameters was achieved by imposing excluded restrictions on the structural equations. Madalla (21) suggested omission of at least one explanatory variable in the reduced-form equations from the structural equation as a predictor variable. Previous applications of this model to health, commonly excluded socioeconomic indicators from the structural equation [e.g., (22–24, 26)]. We follow this approach, assuming that socioeconomic characteristics only indirectly affect disease incidence through lifestyles.

## Results

Tables 2–4 present estimation results from the multivariate probit specifications of the full recursive model in Equation (2) for the whole sample, female and male sub-samples, respectively. We tested the null hypothesis *H*_*o*_*:* ρ = *0* via likelihood ratio test, that the error terms of the equations were jointly statistically equal to zero. This test rejected the null hypothesis of exogeneity, suggesting that estimation of the equations independently of one another would be inefficient.

**Table 2:** Recursive multivariate probit regression: Whole sample.

	**Smoking**	**Drinking**	**Inactivity**	**Diet**	**SMRpost***^a^*****
**Variables**	**Coefficient** ***^b^***	**Coefficient**	**Coefficient**	**Coefficient**	**Coefficient**
**Characteristics:**					
Gender (male)	0.073***	0.523***	−0.093***	0.212***	0.048
Age	0.040***	0.013***	0.0065*	0.0027	0.016***
Age squared	−0.00058***	−0.00021***	0.00015***	−0.00011**	0.00016***
Critical age (Years)***^c^***	34.5***	30.8***	21.8	13.1	51.4*
**Marital status:*^d^***					
Married/cohabiting	−0.208***	−0.162***	−0.022	−0.052	
Divorced widowed/separated	0.145***	−0.101***	−0.051	0.081*	
**Education:*^e^***					
Low level	−0.051*	0.123***	−0.390***	−0.029	
Low middle level	−0.278***	0.225***	−0.336***	−0.200***	
Upper middle level	−0.271***	0.115***	−0.416***	−0.249***	
Degree/professional qual.	−0.451***	0.231***	−0.509***	−0.516***	
Education (missing)	0.133*	−0.107	−0.657***	−0.297***	
**Occupational social status:*^f^***					
Partly skilled manual	−0.054	0.114**	0.173**	−0.087**	
Skilled manual	−0.176***	0.166***	0.237***	−0.339***	
Skilled non-manual	−0.264***	0.110**	0.106**	−0.343***	
Intermediate/managerial	−0.260***	0.216***	0.151***	−0.470***	
Professional	−0.395***	0.180***	0.040	−0.544***	
Skill (missing)	−0.238***	−0.156**	0.064	−0.117	
**Deprivation:*^g^***					
Deprived = 2	0.036	−0.094***	0.094***	−0.030	
Deprived = 3	0.166***	−0.122***	0.185***	0.009	
Deprived = 4	0.274***	−0.059*	0.171***	0.092**	
Most deprived	0.445***	−0.066*	0.248***	0189***	
**SAH and precondition:*^h^***					
SAH (Good)	0.260***	0.055**	0.058***	107***	0.079***
SAH (Fair)	0.467***	0.029	0.198***	0.225***	0.208***
SAH (bad/very bad)	0.656***	−0.0647	0.580***	0.172***	0.515***
Precondition***^i^***	−0.263***	−0.0025	0.350***	−0.229***	0.264***
SMRpre***^j^***	−0.110***	−101**	0.055	−0.064	0.648***
**Endogenous risky behaviours:**					
Smoking					1.086***
Drinking					0.034
Inactivity					0.017
Inadequate diet ***^k^***					0.373***
Constant	−0.763***	−1.168***	−0.831***	1.884***	−3.067***
LR test of Rho's ***^l^***: $\chi_{\left(10\right)}^{2}$	545.5, Prob > $\chi_{\left(10\right)}^{2}$ = 0
Log partial likelihood	−49,521.4				
Observations	20,332	20,332	20,332	20,332	20,332

a *SMRpost, first incidence of a risk related behaviour post survey*.

b The asterisks: ^***^, ^**^, and ^*^ indicate statistical significance at the p < 0.01, p < 0.05, p < 0.1, respectively.

c Critical age, (${\hat{\beta }}_{age}/2*\left({\hat{\beta }}_{agesq}\right)$)in absolute value terms.

d *Reference, Single*.

e *Reference, No educational qualification, Low level, School leaving, CSE, etc., Low middle level, SVQ, “A” levels, OND, etc., Upper middle level, City and Guilds, Higher National Diploma, etc*.

f *Reference, Unskilled/others*.

g *Reference, Least deprived (Deprived = 1)*.

h *SAH, Self-assessed health, reference, “Very good”*.

i *Preconditions include IHD, Type 2 diabetes, treatment for high blood pressure, limiting and non-limiting long-standing illness, hospital attendance for a cardiovascular disease, etc*.

j *SMRpre, 1 if the individual has a behaviour related diseases incidence post-survey*.

k *Inadequate diet is defined in terms of not meeting the recommended five portions a day fruit and vegetable consumption*.

l *Likelihood Ratio test of the null hypothesis that the covariance parameters (ρ_jk_) are jointly statistically not different from zero*.

**Table 3:** Recursive multivariate probit regression: Females.

	**Smoking**	**Drinking**	**Inactivity**	**Diet**	**SMRpost***^a^*****
**Variables**	**Coefficient** ***^b^***	**Coefficient**	**Coefficient**	**Coefficient**	**Coefficient**
**Characteristics:**					
Age	0.038***	0.0091	−0.0102**	−0.010	0.014***
Age squared	−0.00057***	−0.00021***	0.0003***	1.00e-05	0.00014**
Critical age (Years)***^c^***	33.0***	21.9***	17.7***	49.5	60.0
**Marital status^d^**					
Married/cohabiting	−0.221***	−0.160***	−0.004	−0.059	
Divorced/ widowed/separated	0.138***	−0.241***	−0.026	0.017	
**Education:^e^**					
Low level	−0.053	0.117***	−0.345***	−0.039	
Low middle level	−0.370***	0.221***	−0.393***	−0.242***	
Upper middle level	−0.318***	0.030	−0.518***	−0.162***	
Degree/professional qual.	−0.535***	0.346***	−0.521***	−0.537***	
Education (missing)	−0.0222	−0.463***	−0.785***	−0.269	
**Occupational social status:^f^**					
Partly skilled manual	−0.036	0.172**	0.169***	−0.148	
Skilled manual	−0.169***	0.144*	0.308***	−0.385***	
Skilled non-manual	−0.285***	0.234***	0.079	−0.300***	
Intermediate/managerial	−0.216***	0.332***	0.162***	−0.496***	
Professional	−0.433***	0.290***	0.133	−0.629***	
Skill (missing)	−0.128*	0.088	0.057	−0.124	
**Deprivation: ^g^**					
Deprived = 2	0.0017	−0.107**	0.080*	−0.023	
Deprived = 3	0.207***	−0.193***	0.188***	−0.029	
Deprived = 4	0.280***	−0.187***	0.173***	0.095*	
Most deprived	0.436***	−0.170***	0.242***	0.144**	
**SAH and precondition:^h^**					
SAH (Good)	0.232***	0.039	0.017	0.069*	0.130***
SAH (Fair)	0.426***	−0.088*	0.125***	0.176***	0.227***
SAH (bad/very bad)	0.543***	−0.163**	0.541***	0.140*	0.568***
Precondition***^i^***	−0.226***	0.073**	0.320***	−0.187***	0.280***
SMRpre***^j^***	−0.071	−0.108	0.118*	−0.052	0.664***
**Endogenous risky behaviours:**					
Smoking					1.007***
Drinking					−0.463**
Physically Inactive					−0.031
Inadequate diet***^k^***					0.414*
Constant	−0.658***	−0.997***	−0.359***	2.245***	−2.541***
LR test of Rho's ***^l^***: $\chi_{\left(10\right)}^{2}$	chi2(10) = 326.98, Prob > $\chi_{\left(10\right)}^{2}$ = 0				
Log partial likelihood	−26,519.76				
Observations	11,169	11,169	11,169	11,169	11,169

a *SMRpost, first incidence of a risk related behaviour post survey*.

b The asterisks: ***, **, and * indicate statistical significance at the p < 0.01, p < 0.05, p < 0.1, respectively.

c Critical age, (${\hat{\beta }}_{age}/2*\left({\hat{\beta }}_{agesq}\right)$)in absolute value terms.

d *Reference, Single*.

e *Reference, No educational qualification, Low level, School leaving, CSE, etc., Low middle level, SVQ, “A” levels, OND, etc., Upper middle level, City and Guilds, Higher National Diploma, etc*.

f *Reference, Unskilled/others*.

g *Reference, Least deprived (Deprived = 1)*.

h *SAH = Self-assessed health, reference, “Very good”*.

i *Preconditions include IHD, Type 2 diabetes, treatment for high blood pressure, limiting and non-limiting long-standing illness, hospital attendance for a cardiovascular disease, etc*.

j *SMRpre, 1 if the individual has a behaviour related diseases incidence post-survey*.

k *Inadequate diet is defined in terms of not meeting the recommended five portions a day fruit and vegetable consumption*.

l *Likelihood Ratio test of the null hypothesis that the covariance parameters (ρ_jk_) are jointly statistically not different from zero*.

**Table 4:** Recursive multivariate probit regression: Males.

	**Smoking**	**Drinking**	**Inactivity**	**Diet**	**SMRpost***^a^*****
**Variables**	**Coefficient** ***^b^***	**Coefficient**	**Coefficient**	**Coefficient**	**Coefficient**
**Characteristics**:					
Age	0.041***	0.014**	0.026***	0.019**	0.017***
Age squared	−0.00058***	−0.0002***	−2.52e-05	−0.00026***	0.00019***
Critical age (Years)***^c^***	35.4***	35.3***	50.7	37.1***	44.2
**Marital status:^d^**					
Married/cohabiting	−0.214***	−0.134***	−0.054	−0.052	
Divorced/ widowed/separated	0.135***	0.110**	−0.061	0.228***	
**Education:^e^**					
Low level	−0.052	0.103**	−0.465***	−0.039	
Low middle level	−0.177***	0.198***	−0.294***	−0.171**	
Upper middle level	−0.223***	0.180***	−0.328**	−0.351***	
Degree/professional qual.	−0.365***	0.118**	−0.505***	−0.514***	
Education (missing)	0.309***	0.091	−0.501***	0.324	
**Occupational social status:^f^**					
Partly skilled manual	−0.086	0.0541	0.165**	−0.281*	
Skilled manual	−0.202***	0.111	0.179**	−0.335**	
Skilled non-manual	−0.266***	−0.0413	0.166**	−0.507***	
Intermediate/managerial	−0.313***	0.103	0.130*	−0.466***	
Professional	−0.407***	0.113	−0.053	−0.506***	
Skill (missing)	−0.424***	−0.439***	−0.0015	−0.130	
**Deprivation:^g^**					
Deprived = 2	0.0728	−0.089**	0.110**	−0.038	
Deprived = 3	0.110***	−0.064	0.186***	0.066	
Deprived = 4	0.260***	0.0432	0.167***	0.073	
Most deprived	0.446***	0.016	0.246***	0.242***	
**SAH and precondition:^h^**					
SAH (Good)	0.290***	0.081**	0.109***	0.156***	0.021*
SAH (Fair)	0.510***	0.134***	0.294***	0.295***	0.179***
SAH (bad/very bad)	0.793***	0.0068	0.624***	0.215**	0.404***
Precondition***^i^***	−0.303***	−0.057*	0.393***	−0.279***	0.293***
SMRpre***^j^***	−0.167***	−0.108*	−0.046	0.075	0.596***
**Endogenous risky behaviours:**					
Smoking					1.290***
Drinking					−0.261
Physically Inactive					−0.181
Inadequate diet***^k^***					0.861***
Constant	−0.746***	−0.722***	−1.451***	1.701***	−3.139***
LR test of Rho's ***^l^***: $\chi_{\left(10\right)}^{2}$	Chi2(10) = 218.51, Prob > $\chi_{\left(10\right)}^{2}$ = 0				
Log partial likelihood	−22,749.8				
Observations	9,163				9,163

a *SMRpost, first incidence of a risk related behaviour post survey*.

b The asterisks: ***, **, and * indicate statistical significance at the p < 0.01, p < 0.05, p < 0.1, respectively.

c Critical age, (${\hat{\beta }}_{age}/2*\left({\hat{\beta }}_{agesq}\right)$)in absolute value terms.

d *Reference, Single*.

e *Reference, No educational qualification, Low level, School leaving, CSE, etc., Low middle level, SVQ, “A” levels, OND, etc., Upper middle level, City and Guilds, Higher National Diploma, etc*.

f *Reference, Unskilled/others*.

g *Reference, Least deprived (Deprived = 1)*.

h *SAH, Self-assessed health, reference, “Very good”*.

i *Preconditions include IHD, Type 2 diabetes, treatment for high blood pressure, limiting and non-limiting long-standing illness, hospital attendance for a cardiovascular disease, etc*.

j *SMRpre, 1 if the individual has a behaviour related diseases incidence post-survey*.

k *Inadequate diet is defined in terms of not meeting the recommended five portions a day fruit and vegetable consumption*.

l *Likelihood Ratio test of the null hypothesis that the covariance parameters (ρ_jk_) are jointly statistically not different from zero*.

The first four columns in Tables 2–4 present the partial effects for the reduced form risk behaviour equations. In Table 2 for the whole sample, males were strongly more likely than females to undertake risky behaviours; smoking, excessive drinking and inadequate diet. These results agree with the findings from German data by Schneider and Schneider (26). However, males were less likely than females to be physically inactive.

The age effect differed across health risky behaviours and between the estimated models. Generally, age had a quadratic effect on the probability of smoking and excessive drinking. Here, the function was inverted U-shaped, suggesting that the probability of smoking and drinking excessively was increasing with age initially, and then decreased. The critical age (in years) at which this change occurred differed across the estimated models, but was statistically significant at 5% or better. For the whole sample, the critical age at which the probability of smoking began to diminish was 34.5 years, compared to 30.8 years for excessive drinking. However, the diminishing smoking and excessive drinking probabilities occurred at a relatively higher age for males (Table 4) than for females (Table 3). For males, the diminishing effect occurred at around aged 35 years on average, compared to aged 33 years and ~22 years for females, respectively.

The age effect for physical inactivity and inadequate diet differed between the whole sample and by gender. For the whole sample, the probability of inactivity and inadequate diet was increasing monotonically and the critical ages were statistically insignificant (Table 2). The functional form of the effect of age on physical inactivity and inadequate diet differed markedly between females (Table 3) and males (Table 4). For the female sample, the functional forms for physical inactivity and inadequate diet were U-shaped, respectively. This suggests an increasing effect of age on these health risky behaviours. Thus, the critical age at which physical inactivity increased amongst females was strongly statistically significant at ~18 years, but insignificant for inadequate diet. For the male sample however, age has a diminishing effect on inadequate diet with an inverted U-shaped function and the critical age at which inadequate diet decreased amongst males was statistically significant at ~37 years, but insignificant for physical inactivity.

Regarding marital status, married or cohabiting individuals were less likely than singles to undertake all the health risky behaviours across all models, except physical inactivity and inadequate diet, in which the negative effect was statistically insignificant (Table 2). Divorced, separated or widowed women were more likely to be smokers than singles and less likely to drink excessively (Table 3). For men however, being divorced, separated or widowed was positively associated with smoking, excessive drinking and inadequate diet (Table 4). For both females and males, marital status had no effect on physical inactivity.

For education, the results suggested that whilst the more educated were less likely than those with no education to be smoking, physically inactive and taking inadequate diet, they were also more likely to drink excessively. The result on drinking is consistent with findings by Schneider and Schneider (26), in which the result was associated with social acceptance of alcohol consumption amongst the more educated. The results for education attainment were consistent across all estimated models.

The effect of occupational social status also differed across the health risky behaviours and by gender. Generally, and particularly for the whole and female sample, smoking and inadequate diet probability decreased progressively with higher occupation levels, whilst the probability of excessive drinking and physical inactivity increased generally with higher occupational status (Tables 2, 3).^2^

Similarly for males (Table 4), the probability of smoking and inadequate diet decreased progressively with higher occupational status, whilst the probability of being physical inactive increased. In contrast to females, the effect of occupational status on the probability of excessive drinking was not statistically different from zero. Overall, these results suggested that whilst the more skilled individuals were less likely to smoke or have an inadequate diet, they were more likely than those with no skills to be physically inactive, particular females. Results for excessive drinking were less clear-cut.

The deprivation effect also differed across health risky behaviours and by gender. The most deprived females (SIMD55) were more likely than females in the least deprived quintile (SIMD51) to be smoking, physically inactive, and had an inadequate diet, but were less likely to drink excessively.^3^ Males in the most deprived quintile were also more likely than males in the least deprived quintile to be smoking, physically inactive, and taking inadequate diet. There was no statistical evidence supporting the influence of deprivation on excessive drinking.^4^, ^5^

Generally, the probability of smoking, physical inactivity, and inadequate diet was positively associated with worse self-assessed general health (Table 2). However, the relationship between health risky behaviours and self-assessed general health also differed by gender. For females, the probability of smoking, physical inactivity, and inadequate diet was positively associated with worse self-assessed general health, whilst the probability of excessive drinking was negatively associated with worse self-assessed general health.

Further, having a self-reported precondition decreased the probability of smoking and inadequate diet, but increased the probability of physical inactivity in all samples. However, females who reported a precondition were also more likely to drink excessively whilst for males, a precondition reduced the likelihood of excess drinking. Additionally, a pre-survey disease incidence (SMRpre) decreased smoking and drinking probability in the whole sample and for males. For females however, a pre-survey disease incidence was only statistically associated with physical inactivity.

The last columns in Tables 2–4 present results for the structural disease incidence equation. For the whole sample, there was no statistical evidence of gender differences in the probability of a behaviour related disease incidence. As expected, the probability of a behaviour-related disease incidence increased monotonically with age across all estimated models, but the critical age was only statistically significant for the whole sample.

For the health-related characteristics, those in worse self-assessed health were strongly more likely than those assessing their heath as “Very Good”, to suffer a behaviour-related disease incidence post-survey. The probability of a disease incidence increased progressively with worsening self-assessed health. Experiencing a behaviour-related disease incidence pre-survey or self-reported pre-condition was positively associated with experiencing a disease incidence post-survey.

For endogenous risk behaviour variables, it should be noted that the estimates were conditional on the individual characteristics influencing each of the health risk behaviours. Therefore, the estimates were the direct and indirect effects on disease incidence. There was a gender difference in the impact of excessive drinking on the probability of a disease incidence post-survey, which was negatively associated for females and insignificant for males. For males and females, smoking and inadequate diet showed a positive statistically significant effect on the probability of disease incidence post-survey whilst physical inactivity was statistically insignificant.

Finally, we assessed dependency between the estimated equations and tested whether the health risky behaviours were endogenous to disease incidence. Table 5 presents the results. Each sub-table reports the pairwise correlations between the health behaviours and disease incidence and the correlation coefficients ρ_*jk*_.^6^ The results showed that most health risk behaviours were positively correlated and coefficients were highly statistically significant, except in the males sample, where the correlation between smoking and inactivity (ρ_*smoking* * *inactive*_) and between drinking and inadequate diet were insignificant.

**Table 5:** Correlation matrices for the restricted and the full model^a^.

	**Smoking**	**Drinking**	**Inactivity**	**Diet**	**Incidence**
**Whole sample**					
Smoking	1.00				
	*1.00*				
Excessive drinking	0.141***	1.00			
	0.149***	*1.00*			
Physical inactivity	0.034***	−0.096***	1.00		
	0.055***	*0.005*	1.00		
Inadequate diet	0.318***	−0.061***	0.086***	1.00	
	0.211***	-0.069***	0.118***	*1.00*	
Disease incidence	0.115***	−0.099***	0.308***	0.115***	1.00
	-0.525***	*-0.068*	*-0.063*	-0.287***	*1.00*
**Female sample**					
Smoking	1.00				
	1.00				
Excessive drinking	0.124***	1.00			
	0.183***	*1.00*			
Physical inactivity	0.044***	−0.127***	1.00		
	0.060***	*-0.008*	*1.00*		
Inadequate diet	0.307***	−0.153***	0.083***	1.00	
	0.195***	-0.121***	0.109***	*1.00*	
Disease incidence	0.091***	−0.208***	0.269***	0.108***	1.00
	-0.480***	*0.091*	*0.108*	-0.277***	*1.00*
**Male sample**					
Smoking	1.00				
	*1.00*				
Excessive drinking	0.158***	1.00			
	0.120***	*1.00*			
Physical inactivity	0.025	−0.051***	1.00		
	0.045**	*0.009*	*1.00*		
Inadequate diet	0.333***	−0.004	0.095***	1.00	
	0.239***	*-0.012*	0.123***	*1.00*	
Disease incidence	0.146***	−0.033*	0.362***	0.125***	1.00
	-0.597***	*0.151*	*0.068*	-0.367***	*1.00*

a *The figures for the full models are shown in italics*.

The asterisks: ***, **, and * indicate statistical significance at p < 0.01, p < 0.05, p < 0.1, respectively.

These results suggested that unobserved factors influencing the likelihood of one risky health behaviour also contribute positively to the likelihood of other health risky behaviours and disease incidence. Secondly, only smoking and unhealthy diet were significantly negatively correlated with a disease incidence. This suggested that these healthy risky behaviours were endogenous to disease risk and should be modelled jointly with disease incidence. These general patterns of correlation were observed across all estimated models.

However, this study is without limitations. Firstly, our modelling approach did not account for potential sources of endogeneity bias. Specifically, self-assessed general health included as an explanatory variable may be jointly determined with the health outcomes. Also, the exclusion of year dummies from the model constrained an understanding of whether the year of survey predicted the outcomes. Future research should consider these limitations in modelling approach.

## Discussion

This study was motivated by the need to understand the interrelationships between health risky behaviours and their effect on related health outcomes such as disease incidence. This was considered crucial in designing behaviour intervention programmes. Incidence of many of the main diseases and premature deaths tend to be influenced by multiple health risky behaviours, such as those considered in this study. Risky behaviours tend to be dependent and they may have a multiplicative effect on disease incidence and healthcare cost.

We examined interrelationships between health risky behaviours smoking, excessive drinking, physical inactivity and inadequate diet and associated disease outcomes, using linked healthcare data on Scottish adults aged 16 and above. Specifically, we sought to assess the demographic, socioeconomic, and other factors associated with health risky lifestyle choices and to investigate whether or not these risky lifestyle choices have a significant influence on the probability of having a behaviour-specific disease incidence.

The study adds to the literature on the modelling of the determinants of multiple health behaviours, particularly risky behaviours, and how these determinants might differ by gender. This was made possible by use of a unique data set linking individual characteristics directly to their hospitalisation episodes. This avoided biases often associated with wholly self-reported data.

A key advantage of using linked healthcare dataset was that the record linkage provided a way to approximate causality since the health outcome was behaviour-specific. Also, the modelling strategy employed enabled examination of associations between respondent characteristics and each health risky behaviour, and the overall direct and indirect effects they have on disease incidence. Moreover, focusing on health risky behaviours rather than health protective behaviours was premised on the growing evidence that targeting multiple risky behaviours rather than a single risky behaviour at a time, offers the potential for maximising health promotion and intervention benefit, and reducing costs associated with behaviour-related disease outcomes [e.g., (9, 29)].

Understanding the interrelationships between risky health behaviours and associated disease outcomes is useful for intervention design aiming to promote multiple risky behaviour change at a time. This understanding is based on potential spillover effects arising when improvement in one behaviour increases the likelihood of adopting other healthier lifestyle behaviours.^7^ That is; a beneficial single behaviour change may incentivize adoption of another healthy behaviour (30).

The results showed clear demographic and socioeconomic gradients in the risky behaviour determinants, particularly smoking, excessive drinking and inadequate diet, but are less clear cut for physical inactivity. Gender differences in the observed characteristics influencing risky behaviours and disease outcome were found. Undertaking risky behaviours increased generally at younger ages and tended to decrease as one becomes older. However, males were more likely than females to undertake risky behaviours longer in their lives, such as smoking, excessive drinking and inadequate diet. The estimate of the critical ages suggested that physical inactivity amongst females could begin as early as <13 years of age.

Our findings also support education as a key driver of better health behaviour irrespective of gender. A social gradient in healthy risk behaviours was also found in the analysis given that our findings suggested that a lower social standing, as measured by deprivation, induced health risky behaviour which increased the probability of associated disease incidence.

Moreover, we found that disease incidence was positively correlated with smoking and inadequate diet, whilst these same lifestyles were negatively correlated with having a precondition. This an important result suggesting that having a pre-condition may motivate healthier lifestyles (in terms of reduction in smoking and inadequate diet) in the future. It would be of interest to understand how these results generalise into other populations. In summary, demographic and socioeconomic characteristics such as age, gender, marital status, education, the environment, and having a precondition were some of the key elements influencing health risky behaviours and indirectly behaviour-related health outcome. Hence, designing intervention programmes targeting health risky behaviours may be more beneficial by greater investments in education and improving the environment to be more conducive for the younger age groups. Improvement in the modelling approach in future research will address the limitations.

## Data Availability Statement

The datasets presented in this article are not readily available because permission to access to the Linked SheS dataset used in this study can be granted by application to the Privacy Advisory Committee, Information Services Division of the NHSScotland. Requests to access the datasets should be directed to https://www.isdscotland.org/.

## Author Contributions

DO: conceptualisation, econometric modelling and analysis, and initial draught preparation and writing. BE: conceptualisation, analytical support, literature review, and review and editing. AL: conceptualisation, literature review, supervision, and review and editing. All authors have read and agreed to the final draft of the manuscript.

## Funding

The receipt of financial support from the MRC National Preventive Research Initiative Phase 2 Grant G0701874 is greatly acknowledged. The Funding Partners relevant to this award are: British Heart Foundation, Cancer Research UK, Department of Health, Diabetes UK, Economic and Social Research Council, Medical Research Council, Research and Development Office for the Northern Ireland Health and Social Services, Chief Scientist Office, Scottish Government Health Directorates, The Stroke Association, Welsh Assembly Government, and World Cancer Research Fund. The Health Economics Research Unit is funded by the Chief Scientist Office of the Scottish Government Health Directorates. The views expressed in this article are those of the authors.

## Conflict of Interest

The authors declare that the research was conducted in the absence of any commercial or financial relationships that could be construed as a potential conflict of interest.

## Publisher's Note

All claims expressed in this article are solely those of the authors and do not necessarily represent those of their affiliated organizations, or those of the publisher, the editors and the reviewers. Any product that may be evaluated in this article, or claim that may be made by its manufacturer, is not guaranteed or endorsed by the publisher.

## Footnotes

^1^The limit for men has since been reduced to the same level as for women.

^2^Statistically insignificance was based on joint tests post-estimation, which gave Chi squared estimates and associated p-value.

^3^For inadequate diet, the coefficient were statistically significant only at the 4th and the 5th quintiles. However, they were jointly significant: Chi2(4) = 14.08, p-value = 0.007.

^4^For excessive drinking, only the coefficient on the 2nd quintile is statistically significant and a joint significance test of the quintiles cannot reject the null hypothesis that the effect of deprivation on excessive drinking is not different from zero: Chi2(4) = 13.16, p-value = 0.105.

^5^For inadequate diet, only the coefficient on the 5th quintile is statistically significant and a joint significance test suggests that the quintiles are jointly significant: Chi2(4) = 16.66, p-value= 0.0023.

^6^In estimation, the statistic $z\;=\;\hat{\rho }/s_{\hat{\rho }}$ was computed, where was the asymptotic standard error of $\hat{\rho }$.

^7^The idea of spillover effect here is synonymous with the concept of co-variation amongst multiple health behaviours in the literature on preventive medicine [e.g. (29)].
